# Cationic and PEGylated Amphiphilic Cyclodextrins: Co-Formulation Opportunities for Neuronal Sirna Delivery

**DOI:** 10.1371/journal.pone.0066413

**Published:** 2013-06-21

**Authors:** Aoife M. O’Mahony, Julien Ogier, Raphael Darcy, John F. Cryan, Caitriona M. O’Driscoll

**Affiliations:** 1 Pharmacodelivery group, School of Pharmacy, University College Cork, Cork, Ireland; 2 School of Chemistry and Chemical Biology, University College Dublin, Dublin, Ireland; 3 Dept. of Anatomy and Neuroscience, University College Cork, Cork, Ireland; University of Helsinki, Finland

## Abstract

Optimising non-viral vectors for neuronal siRNA delivery presents a significant challenge. Here, we investigate a co-formulation, consisting of two amphiphilic cyclodextrins (CDs), one cationic and the other PEGylated, which were blended together for siRNA delivery to a neuronal cell culture model. Co-formulated CD-siRNA complexes were characterised in terms of size, charge and morphology. Stability in salt and serum was also examined. Uptake was determined by flow cytometry and toxicity was measured by MTT assay. Knockdown of a luciferase reporter gene was used as a measure of gene silencing efficiency. Incorporation of a PEGylated CD in the formulation had significant effects on the physical and biological properties of CD.siRNA complexes. Co-formulated complexes exhibited a lower surface charge and greater stability in a high salt environment. However, the inclusion of the PEGylated CD also dramatically reduced gene silencing efficiency due to its effects on neuronal uptake. The co-formulation strategy for cationic and PEGylated CDs improved the stability of the CD.siRNA delivery systems, although knockdown efficiency was impaired. Future work will focus on the addition of targeting ligands to the co-formulated complexes to restore transfection capabilities.

## Introduction

Silencing of genes using RNA interference (RNAi) based technology is one of the most exciting areas of research in modern molecular medicine [1]–[3]. Progression of RNAi towards *in vivo* use requires a greater focus on overcoming the stability and targeting issues associated with cationic siRNA delivery vectors [4], [5]. In particular, improving delivery of siRNA to neurons and the CNS remains a challenge [6], [7].

**Figure 1:**
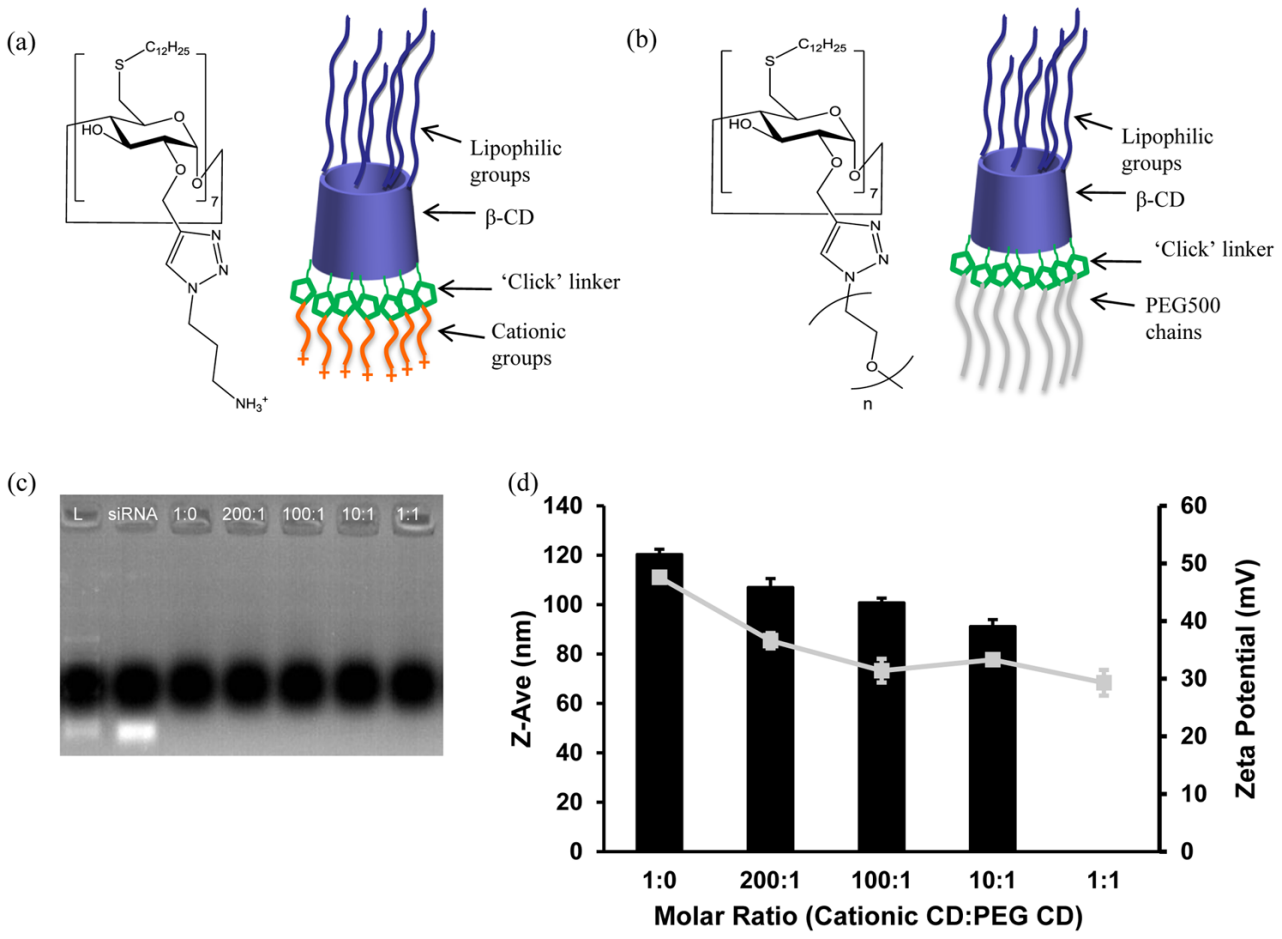
Chemical structures and properties of CDs and complexes. (a, b) Chemical structures and schematic representations of Cationic CD (a) and PEGylated CD (n = 10–12) (b). (c) Gel electrophoresis showing siRNA binding within co-formulated CD.siRNA complexes. (d) Size (Z-Ave (nm), black bars) and charge (zeta potential (mV), grey square boxes) of co-formulated CD.siRNA complexes. Data are presented as the Mean ± S.D (n = 3).

Cyclodextrins (CDs) modified with various amphiphilic and cationic groups offer great potential as non-viral gene and siRNA delivery vectors [8]–[10]. Indeed, we have shown successful gene delivery by modified β-CDs to a variety of cell types including liver cells and intestinal epithelial cells and to *in vitro* and *in vivo* tumour models [11]–[15]. A recent development in the modification of CDs is the ‘click’ chemistry based synthesis of a cationic amphiphilic CD, SC12CDClickpropylamine (Fig. 1 (a)) [16]. This CD was effective at mediating siRNA delivery in non-neuronal cells [16] and neuronal cells [17], [18].

An interesting strategy for the optimisation of non-viral vector delivery systems is the ‘ABCD’ nanoparticle concept by Kostarelos and Miller [19]. Here, ‘A’ represents the nucleic acid cargo, ‘B’ represents the component which complexes the nucleic acid, ‘C’ is a stabilising component and ‘D’ is a targeting moiety. Typically, ‘C’ is a polyethyleneglycol (PEG) component, which acts as a steric shield to prevent interaction with plasma proteins, inhibits uptake by the reticulo-endothelial system, improves stability in biological fluids and minimises the toxicity associated with cationic vectors [20]–[22]. The aforementioned cationic click CD-siRNA formulation, effective at mediating transfection in neurons [17], fits the ‘AB’ paradigm. Therefore, to improve this formulation for *in vivo* use, further modifications, including PEGylation, are required.

Commonly used strategies for developing PEGylated nucleic acid vectors include post-insertion of PEGylated lipids into preformed lipoplexes [23] or lipid nanocapsules [24], grafting of PEG chains onto cationic polymers [20], [21] or addition of a PEGylated polymer to pre-formed polymer-siRNA complexes [25]. siRNA itself has also been directly conjugated with PEG chains before complexation by a cationic peptide [26] or Lipofectamine^TM^ 2000 [27] with improvements reported in nuclease stability and immunogenicity, whilst maintaining gene silencing efficiency. Yet another strategy for PEGylation is the mixing of a cationic component with its PEGylated counterpart in a formulation, before complexation of siRNA. This approach has been exploited for both polymeric [22] and lipidic [28] vectors.

Regarding CD-based fomulation strategies, the most widely used approach for PEGylation exploits their ability to form an inclusion complex with guest molecules such as adamantane, via the hydrophobic cavity of the CD molecule [29]. Using this mechanism, PEG chains, chemically linked to adamantane, have been incorporated into CD-containing vectors such as chitosan-PEI-β CD copolymers [30] or cationic CD-containing polymers (CDP) [29], [31]. In another approach, we have reported modifying pre-formed CD.siRNA complexes at their surface by inclusion of a lipid-PEG conjugate in the formulation [32].

Direct modification of the CD structure with PEG chains has proved more challenging. Early approaches by our group included attachment of short oligo(ethylene oxide) moieties at the 2-positions by base-catalysed reaction with ethylene carbonate [11], [33], but this synthesis was not amenable to attachment of longer PEG chain lengths. The recently developed copper-catalysed ‘click’ chemistry approach used for modifying CDs has facilitated selective attachment of PEG chains to the 2-position of an amphiphilic CD [16]. The structure of the resulting PEGylated amphiphilic CD is illustrated in Fig. 1 (b). Hydrocarbon group C12 was chosen based on previous work showing that increasing the chain length of amphiphilic CDs improves transfection efficiency [11], [12], while PEG MW 500 was chosen based on the potential for immunogenicity with higher PEG MW [50].

Previously, we reported preliminary efforts to blend the cationic and PEGylated CDs together in a co-formulation [16]. The PEG containing formulations exhibited altered properties including greater salt-stability but also a lack of gene silencing efficiency, compared to the cationic CD alone [16].

Delivery of siRNA to neurons is particularly challenging, due to their post-mitotic nature and complexity of neuronal networking [6], [34]. Indeed, we found that for the aforementioned cationic CD, SC12CDClickpropylamine, a higher mass ratio of 20 was required to achieve adequate gene silencing in neuronal cells [17], compared to MR 10, which was sufficient in non-neuronal cells [16]. Using MR 20 CD complexes, knockdown of housekeeping and reporter genes (40–60%) was achieved in primary and immortalised neurons, with minimal toxicity compared to Lipofectamine^TM^ 2000 [17].

Here, as part of our efforts towards developing a PEG-stabilised CD vector for neurons and the CNS, we again applied a co-formulation approach, based on the higher MR complexes. To this end, we investigated the effects of PEGylation in the context of neuronal siRNA delivery, in terms of uptake, neuronal viability and knockdown efficiency. Furthermore, we carried out a detailed characterisation of the co-formulated complexes, including the effects of the PEG component on complex morphology and on the serum-induced aggregation.

SC12CDClickpropylamine and SC12CDClickPEG 500 will be referred to hereafter as cationic CD and PEGylated CD respectively.

## Materials and Methods

### Preparation of Vector.siRNA Complexes

#### siRNAs

Negative control (non-silencing: ns) siRNA (sense sequence 5′- UUC UCC GA CGU GUC ACG U), fluorescein labelled siRNA (sense sequence 5′- UUC UCC GAA CGU GUC ACG U, modified with 3′-fluorescein on the sense strand), pGL3 luciferase siRNA (sense sequence 5′- CUU ACG CUG AGU UCG A) and GAPDH siRNA (sense 5′- GGU CGG UGU GAA CGG AUU U) were obtained from Qiagen (California, USA).

#### CD.siRNA complexes

CD.siRNA complexes were prepared as before [16]. Briefly, the two CDs were weighed out and dissolved in chloroform (1 mg/ml), then mixed together in appropriate volumes to give required molar ratios of cationic to PEGylated CD. The solvent was removed with a gentle stream of nitrogen. Aliquots were stored at −20°C until required. For preparation of CD.siRNA complexes, CDs were rehydrated with deionised water (DIW) (final concentration 1 µg/µl), sonicated for one hour at room temperature (RT), mixed with siRNA in an equal volume of water and incubated for 20–30 min at RT. For all experiments, a fixed cationic CD:siRNA MR of 20 was chosen, based on previous results [17]. All formulations, irrespective of the amount of PEGylated CD incorporated, contained the same proportion of cationic CD to siRNA.

### Gel Retardation Assay

siRNA binding was investigated by agarose gel electrophoresis [21]. CD.siRNA complexes were mixed with loading buffer and DIW to a final volume of 20 µl (containing 0.3 µg siRNA). Samples were added to wells in a 1% agarose gel containing SafeView™ (NBS Biologicals Ltd, England) (6 µl/100 mls). Electrophoresis was carried out at 90 V for 20 minutes, with a Tris-borate-EDTA buffer [35]. Bands corresponding to the DNA ladder (100 b.p.) and unbound siRNA were visualised by UV, using the DNR Bioimaging Systems MiniBis Pro and Gel Capture US B2 software.

### Size and Charge Measurements

Particle Z-average size and charge were measured with Malvern’s Zetasizer Nano ZS, using dynamic light scattering (DLS) and electrophoretic mobility measurements respectively. CD.siRNA complexes were prepared and made up to 1 ml with 0.2 µm filtered DIW. Five readings of Z-average size (nm), polydispersity (25°C, measurement angle 170°) and zeta potential (mV) (25°C, measurement angle 12.8°) were taken. For data analysis, the viscosity (0.8872 mPa.s) and refractive index (1.33) of water were used to determine Z-average size.

### Morphology of CD.siRNA Complexes

The morphologies of CD.siRNA complexes were evaluated using transmission electron microscopy (TEM) as previously described [15], [33], [36]. Briefly, cationic CD alone and CD.siRNA complexes (containing 0.5 µg siRNA) were applied to 400 mesh carbon-film copper grids (Agar Scientific) for a couple of minutes. The grids were blotted with filter paper, stained with 2% (w/w) uranyl acetate and incubated overnight. Images were taken using a JEOL 2000 FXII transmission electron microscope (Jel Ltd., Tokyo, Japan).

### Aggregation Studies

The effects of salt-containing medium and serum on the aggregation of the co-formulated CD.siRNA complexes were investigated by incubating complexes in either OptiMEM® transfection medium or foetal bovine serum (FBS) for 24 hours at 37°C [21], [32]. Following this, size measurements were carried out by DLS.

### Cell Culture

A mouse embryonic hypothalamic cell line (*mHypoE N41)* [37] was obtained from tebu-bio (France) and was maintained in Dulbecco’s modified Eagle’s medium (DMEM, Sigma), supplemented with 10% foetal bovine serum (FBS, Sigma) in a humidified 37°C incubator with 5% CO_2_. Cells were seeded in 24 well and 96 well plates at 3.5×10^4^ and 1.5×10^4^ cells per well respectively. This cell line is a useful model for neuronal cells [17].

### Determination of Cell Viability

Cell viability in terms of mitochondrial dehydrogenase activity was determined by the MTT assay [15], [21]. Cells were seeded in 96-well plates 1 day prior to transfection. siRNA (50 and 100 nM) alone, or in CD.siRNA complexes, was diluted in OptiMEM®, then added to cells in serum-containing medium for 24 h. Media was removed and replaced with 100 µl fresh media and MTT (20 µl of a 5 mg/ml solution) for four hours, after which the formazon crystals produced were dissolved in 100 µl DMSO. Absorbance was measured at 590 nm using a UV plate reader. Each experiment was carried out in triplicate. Results were expressed as % cell viability compared to untreated controls.

### Cellular Uptake Experiments

The level of uptake mediated by transfection complexes was assessed by flow cytometry [38]. Fluorescently labelled siRNA (Qiagen) was used for these experiments. Cells were seeded in 24-well plates 1 day prior to transfection. siRNA (50 nM) alone, or in CD.siRNA complexes, was diluted in OptiMEM® and applied to cells in serum-containing medium for 24 hours. Following this, complexes bound to the extracellular surfaces were removed by washing cells with PBS and by incubation with 250 µl of CellScrub buffer for 15 minutes at room temperature [21]. Cells were removed from the wells and prepared for analysis following several washing steps. The fluorescence associated with 10,000 cells was measured with a FACS Caliber instrument (BD Biosciences) and data was analysed using Cell Quest Pro software. Each experiment was carried out in triplicate.

### Knockdown of Luciferase Reporter Gene

Silencing of an exogenous gene was assessed by measuring knockdown of a luciferase reporter plasmid [17]. The pGL3-luc plasmid contains the firefly luciferase gene under the control of an upstream SV40 promoter [21]. Cells were seeded in 24-well plates 1 day prior to transfection, then transfected with pGL3-luciferase (1 µg/well) complexed to Lf2000 (2.5 µl/ µg pDNA), for three hours. Cells were washed twice with PBS prior to siRNA transfection. pGL3-luciferase siRNA (50 nM) alone, or in CD.siRNA complexes, was diluted in OptiMEM® and added to the cells in serum-containing medium. CD.siRNA complexes with negative control siRNA (ns siRNA) were included as controls. After 24 hours, cells were washed with PBS, lysed with 400 µl of 1× Reporter Lysis Buffer (Promega) and frozen at −80°C. Lysate was collected and centrifuged for 5 min at 13000 rpm. A sample of the supernatant (20 µl) was assayed for expression of luciferase by adding to 100 µl of luciferin (Promega) and measuring the light (relative luminescence units (RLU)) produced 10 seconds later in a Junior LB 9059 luminometer (Promega). Total protein levels in each sample were determined by the BCA Protein Assay (Thermo Scientific) to allow normalisation of luciferase activity (RLU per µg protein). Results were expressed as % gene expression relative to ‘untreated’ control samples (which were transfected with luciferase plasmid only but no siRNA and which were thus assigned as 100% luciferase expression). Each experiment was carried out in triplicate.

### Statistics

One-way analysis of variance (ANOVA) was used to compare multiple groups followed by Bonferroni’s post hoc test. Statistical significance was set at *p < 0.05.

## Results

Co-formulated cationic and PEGylated CD.siRNA complexes were prepared as described in the methods, whereby the two CDs were mixed together in appropriate amounts, prior to complexation of siRNA. The mass ratio (MR) of cationic CD to siRNA was kept constant at MR 20. The amount of PEGylated CD in the CD formulation was varied and was expressed as molar ratio of cationic CD to PEGylated CD. For example, molar ratio 1∶0 represents the cationic CD alone, whereas molar ratio 1∶1 represents the formulation with the greatest proportion of PEGylated CD relative to cationic CD.

### Effect of PEGylation on siRNA Complexation and Size and Charge

Co-formulated CD.siRNA complexes were analysed by agarose gel electrophoresis to determine the binding of siRNA (Fig. 1(c)). A band was observed only in the lane corresponding to free siRNA, confirming that the co-formulated complexes were still capable of binding siRNA to the same extent as the cationic CD alone (1∶0).

To investigate the effects of the PEGylated CD on the size and charge, co-formulated CD.siRNA complexes were prepared and their sizes and charges in water were measured using the Malvern ZetaSizer Nano. Complex sizes decreased with the inclusion of PEGylated CD, from 120 ± 2.1 nm at molar ratio 1∶0 to 91 ± 2.8 nm at 10∶1 (Fig. 1 (d)), a finding which is in agreement with our previously published work based on co-formulated complexes at MR 10 [16]. Samples at molar ratio 1∶1 had a PDI of > 0.5, which indicates considerable variation in particle size and these samples were, therefore, excluded from particle size analysis.

Surface charge of complexes also decreased with increasing amounts of PEGylated CD (Fig. 1 (d)), as expected from our previous work [16]. The highly positive charge of the cationic CD alone (+ 47.6 ± 0.5 mV) was reduced to ∼30 mV at molar ratio 100∶1. However, there was no greater decrease in surface charge with increasing proportions of PEGylated CD.

### Morphology of CD.siRNA Complexes

**Figure 2:**
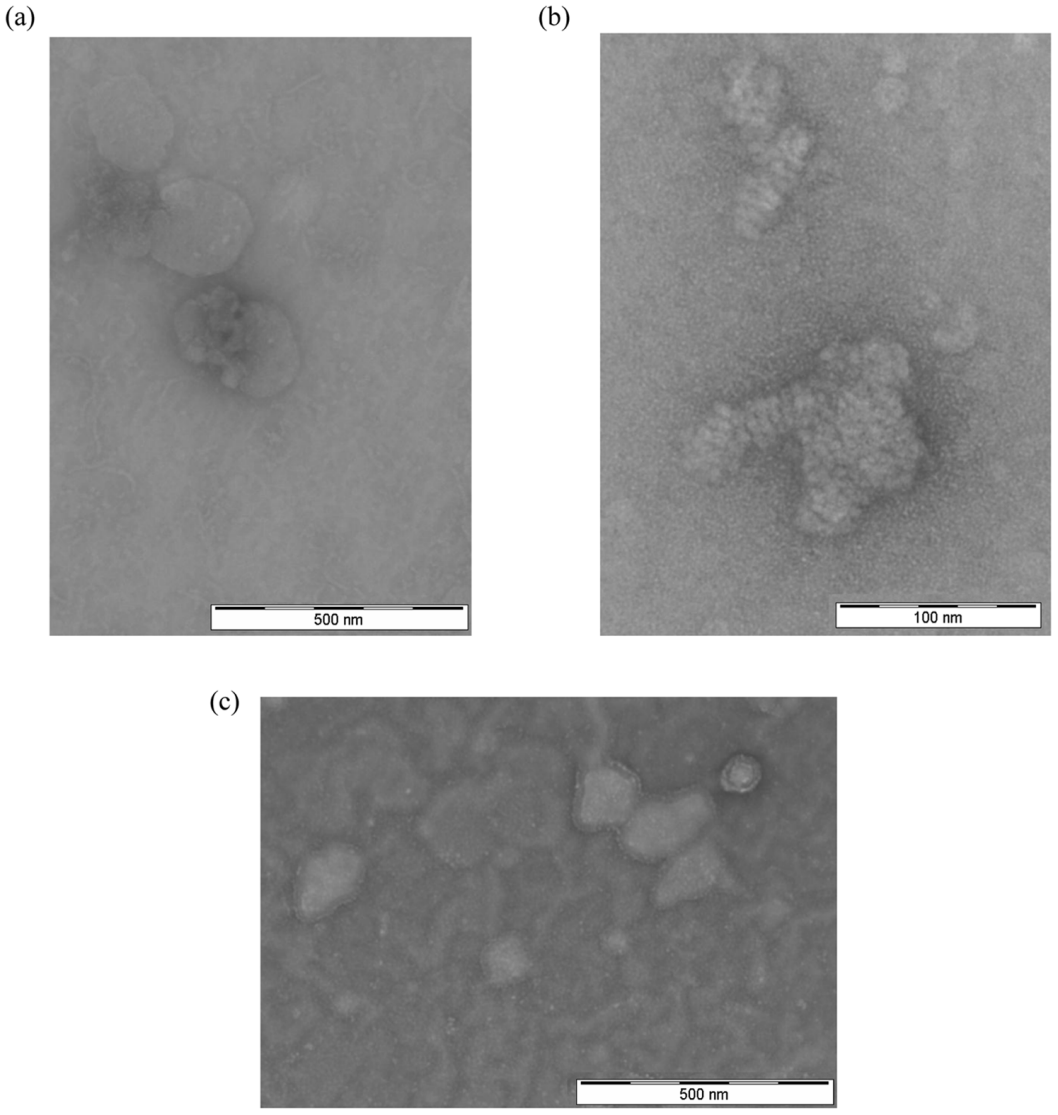
Morphology of CD.siRNA complexes assessed by transmission electron microscopy. (a) Cationic CD alone with no siRNA (magnification: x 60,000) (b) Cationic CD: PEGylated CD Molar ratio 1∶0 (i.e. no PEG) (magnification: x 250,000) and (c) Cationic CD: PEGylated CD Molar ratio 1∶1 (magnification: x 60,000). (Negative staining with 2% uranyl acetate).

The morphologies of CD.siRNA complexes were investigated by TEM, with and without PEGylated CD in the formulation. Generally, the cationic CD (Fig. 2(a)) and its corresponding CD.siRNA complex (Fig. 2(b)) gave irregular shaped particles of 100 – 150 nm in diameter, confirming the particle size measured by DLS. In addition, an irregular looped pattern was observed for CD.siRNA complexes, which was not seen for CD alone. A similar pattern was observed previously for cationic lipid-siRNA lipoplexes [39]. There, a more regular lamellar pattern was shown for some of the lipids investigated, while others gave a pattern which closely resembled that obtained here for the CD.siRNA complexes. For CDplexes with plasmid DNA, a lamellar pattern of alternating DNA and CD layers was previously reported [40].

By comparison, when PEGylated CD was included at molar ratio 1∶1, a dramatic change in the particle morphologies was observed (Fig. 2 (c)), with a distinct ‘shielding’ layer visible around the particles. Moreover, a heterogeneous population made up of individual particles and clusters of particles was evident, confirming the polydisperse nature of this sample.

### Salt- and Serum-induced Aggregation of CD.siRNA Complexes

The conditions to which complexes are exposed *in vitro* or *in vivo* consist of a high salt and serum containing environment [15], [21]. Incorporation of PEG groups into a formulation can confer stability to nanoparticles against salt-induced aggregation and non-specific binding of proteins [41]. Therefore, to investigate these factors, CD.siRNA complexes were incubated with either OptiMEM® (serum-free transfection media containing physiological salt concentrations) or FBS at 37 °C, before measuring particle sizes.

**Figure 3:**
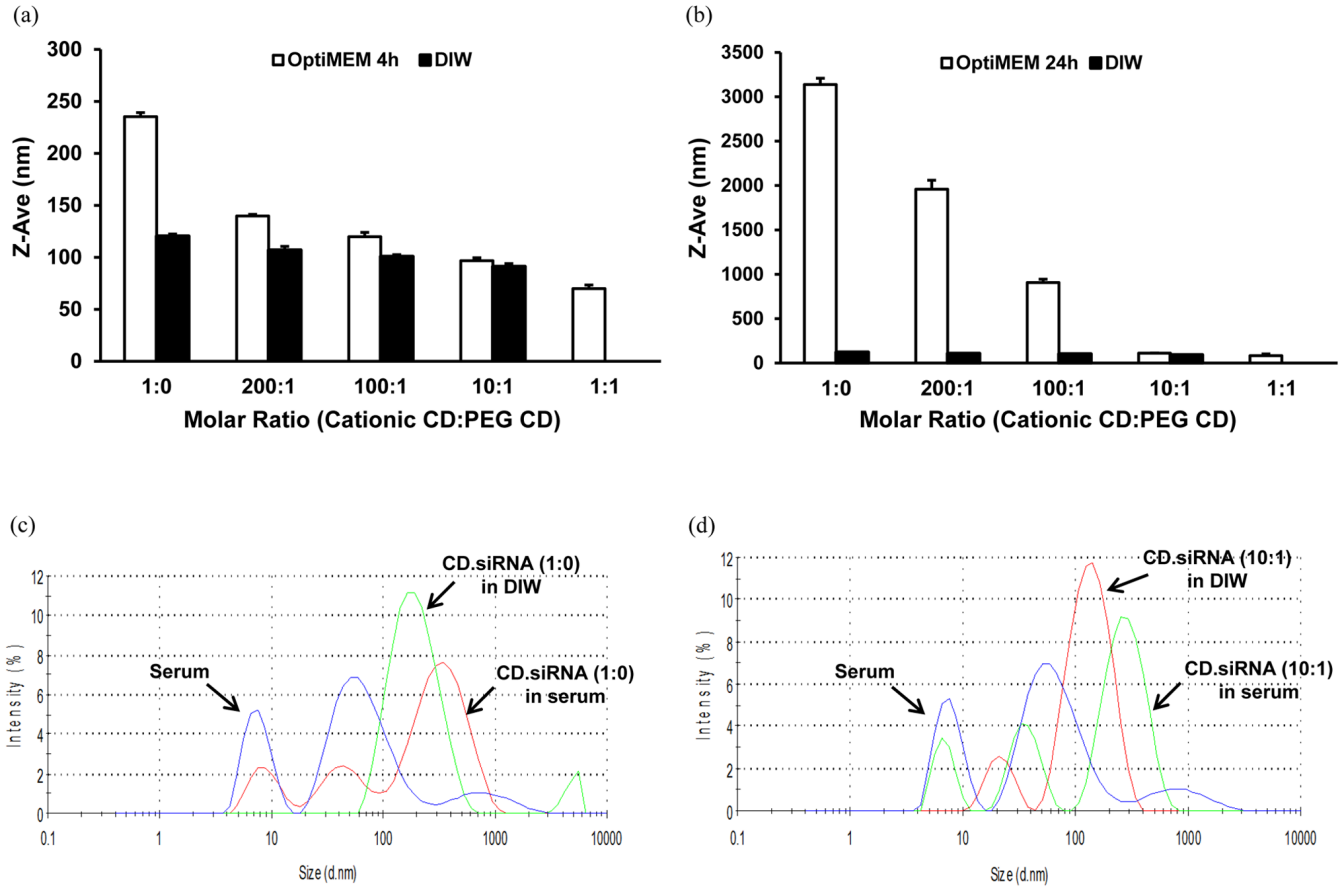
Stability of co-formulated CD.siRNA complexes . Particle size of complexes after incubation in (a, b) OptiMEM® or (c, d) 90% FBS. (a) and (b) depict 4 and 24 hours respectively. (c) and (d) depict co-formulated CD.siRNA complexes at molar ratios 1∶0 and 10∶1 respectively.

CD.siRNA complexes with little or no PEGylated CD in the formulation had the greatest tendency to aggregate in a salt-environment (OptiMEM®) (Fig. 3 (a) and (b)). After four hours incubation, there was a doubling in size of cationic CD.siRNA complexes (Fig. 3 (a)) and after twenty-four hours incubation, large aggregates were present (Fig. 3 (b)). However, those CD.siRNA complexes with PEGylated CD in the formulation showed a marked resistance to aggregation, in particular at molar ratios 10∶1 and 1∶1. This indicates that the PEG component stabilised the complexes and inhibited salt-induced aggregation.

Sizes of CD.siRNA complexes, after incubation in 90% FBS for twenty-four hours, were also investigated. Particle size distribution data generated from DLS measurements demonstrated that significant aggregation occurred when CD.siRNA complexes were incubated in serum, either when the cationic CD was used alone (Fig. 3 (c)) or when the PEGylated CD was included in the formulation (Fig. 3 (d)). This indicates that the PEGylated CD did not stabilise the complexes against non-specific binding of protein [32].

### Effect of PEGylation on Uptake and Gene Silencing Efficiency of CD.siRNA Complexes

**Figure 4:**
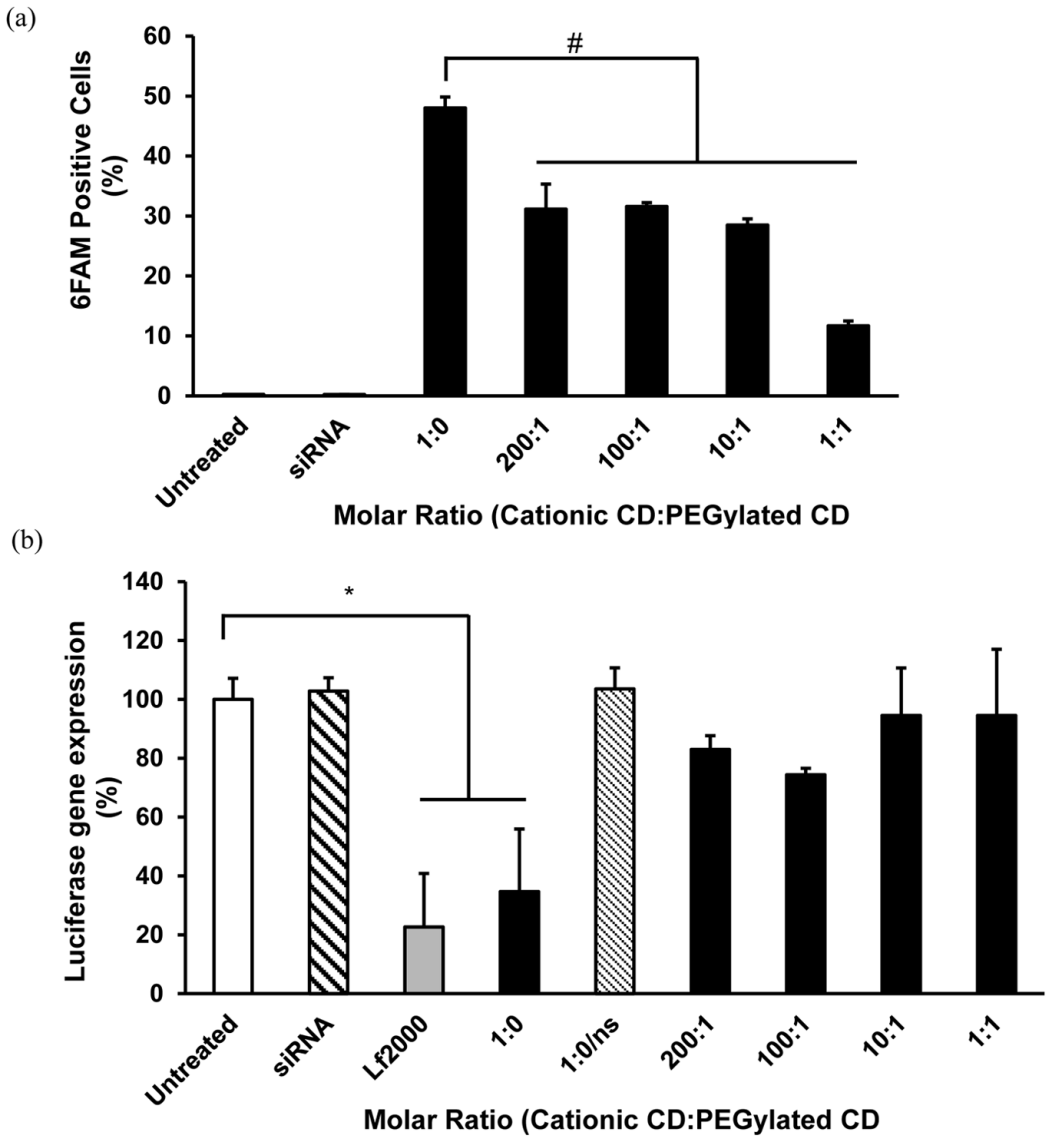
Internalisation and transfection of neuronal cells by CDs. (a) Uptake and (b) Gene knockdown by CD.siRNA complexes in mHypoE N41 cells by co-formulated CD.siRNA complexes (siRNA 50 nM). (a) Complexes were applied for 24 hours before measuring uptake by flow cytometry. Uptake was expressed as percentage of fluorescent siRNA positive cells. (b) Cells were incubated with pGL3-luc complexed to Lf2000 for 3 hours, then CD.siRNA complexes were applied for 24 hours before assessing for luciferase expression. Luciferase expression was calculated as a percentage of untreated cells. Data are presented as mean ± SEM (n = 3). * *p* < 0.05 relative to untreated cells. (1∶0/ns contains non-silencing siRNA and is included as a control).

Fluorescently-labelled siRNA was used to study the uptake of CD.siRNA complexes by neuronal cells, using flow cytometry. High levels of uptake were mediated by the cationic CD.siRNA complexes (i.e. molar ratio 1∶0, no PEGylated CD) (Fig. 4 (a)). However, inclusion of the PEGylated CD in the formulation significantly reduced uptake, at all molar ratios (^#^ *p* < 0.05 compared to 1∶0 CD.siRNA complexes). Uptake was reduced to ∼ 30% at the lowest molar ratio (200∶1). A further decrease to < 12% was observed at molar ratio 1∶1. There was no evidence of internalisation of naked siRNA.

The ability of CD.siRNA complexes to deliver functional siRNA for RNAi was assessed by using luciferase siRNA to silence pGL3 luciferase plasmid expression in neuronal cells. Using 50 nM siRNA, the cationic CD.siRNA complexes (1∶0) achieved a significant reduction in luciferase expression (*p < 0.05 relative to untreated controls), which was comparable to that obtained with Lipofectamine^TM^ 2000 (Fig. 4 (b)). However, inclusion of PEGylated CD completely abolished the gene silencing capabilities of CD.siRNA complexes. Cationic CD complexes containing a control non-silencing siRNA sequence (1∶0/ns) did not affect luciferase expression, confirming specificity of gene knockdown.

### Effect of PEGylation on Toxicity of CD.siRNA Complexes

**Figure 5:**
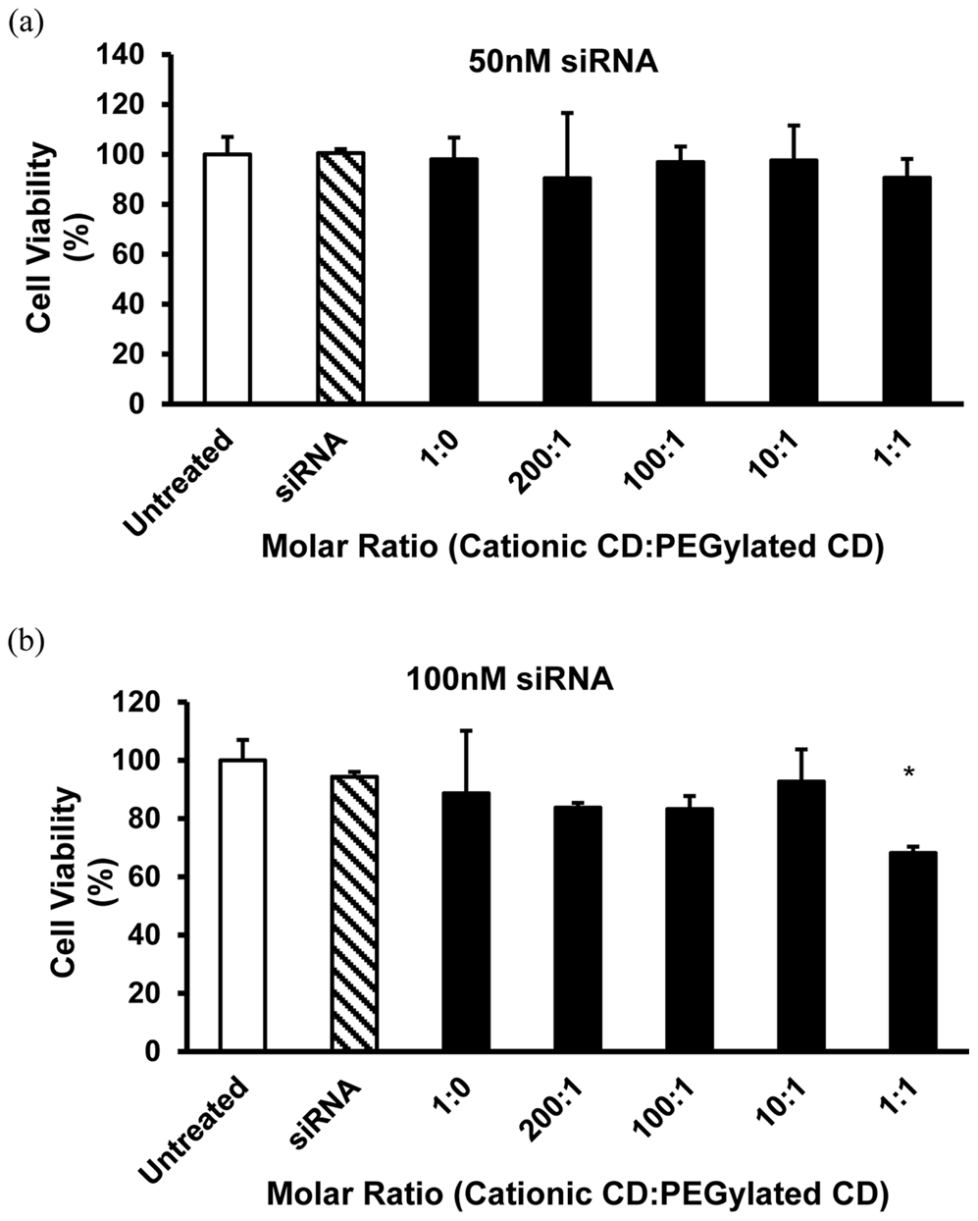
Neuronal viability after treatment with CDs. Viability of mHypoE N41 neuronal cells after treatment with CD.siRNA complexes (MR 20 cationic CD:siRNA) for 24 hours, with increasing content of PEGylated CD in the formulation, (a) 50 nM siRNA and (b) 100 nM siRNA. Cell viability was measured by means of MTT assay. Data are expressed as mean ± SEM (n = 3).

Modification of cationic particles with PEG groups can improve their toxicity profile and, to investigate this, the viability of neuronal cells after 24 hours of treatment with CD.siRNA complexes (50 and 100 nM siRNA), with and without PEGylated CD, was determined by MTT assay. We have previously shown that 100 nM siRNA is required for knockdown of an endogenous housekeeping gene and, therefore, it was important to consider the toxicity of the PEGylated complexes at this higher siRNA concentration [17]. Treatment with cationic CD.siRNA complexes or with mixed CD.siRNA complexes cause minimal toxicity to neuronal cells (Fig. 5). However, at the highest molar ratio (1∶1) and siRNA concentration 100 nM, there was a significant reduction in cell viability (Fig. 5(b), 68 ± 2.2 %, * *p* < 0.05 relative to untreated cells). It is worth noting that, with the exception of the 1∶1 formulation, the levels of toxicity observed here were minimal compared to those obtained with Lipofectamine 2000 (67 ± 4%) in our previous studies [17].

## Discussion

We have previously shown that a modified CD, SC12CDClickpropylamine, was an effective vector for siRNA delivery to neurons [17]. However, due to its cationic nature, this CD is likely to aggregate in the circulation, thereby minimising access to the CNS. Therefore, efforts to improve the formulation towards more favourable properties for *in vivo* use are described here. A co-formulation approach, which involved blending the cationic CD with a PEGylated CD of similar amphiphilicity, was previously investigated in non-neuronal cells (Caco2 cells), for which a lower cationic CD to siRNA MR of 10 was efficient at mediating gene silencing [16]. However, higher MR complexes (MR 20) are required for effective transfection and gene knockdown in neurons [17]. Therefore, we investigated whether the co-formulation approach could be applied to neuronal cells, which are notoriously resistant to transfection and susceptible to the cytotoxic effects of cationic vectors [6], [42].

By applying the ‘ABCD’ structural paradigm for non-viral gene delivery vectors, improved stability and specificity can be achieved by the introduction of additional components such as PEG and/or targeting ligands [19]. Here, we describe ‘ABC’ type nanoparticles, achieved by co-formulating the PEGylated CD (‘C’) with the cationic CD (‘B’) before complexation of siRNA (‘A’), an approach described as ‘pre modification’ [19]. These CDs are compatible in a co-formulation, due to their similar amphiphilicity and this strategy precludes the requirement for additional non-CD based components. The co-formulated CD mixture retained its ability to complex siRNA, as shown in Figure 1 (c). Furthermore, inclusion of the PEGylated CD in the formulation led to a smaller particle size (Fig. 1(d)), as previously reported with other vectors [22], [25] as well as a reduction in surface charge, which was in agreement with our previously studied low MR co-formulation [16]. Changes to the morphologies of CD.siRNA complexes were also apparent (Fig. 2), giving further evidence to successful modification of the complexes with the PEG component.

Furthermore, the PEGylated CD had a dramatic effect on complex stability in the presence of salt-containing medium (OptiMEM®), with little aggregation observed in the 10∶1 co-formulated complexes, even after 24 hours incubation (Fig. 3 (a) and (b)). This is indicative of a PEG layer at the surface of the complexes [30] and is in agreement with previously published data regarding PEGylated CD formulations [16], [32]. However, the PEG component was not sufficient to prevent interaction with serum proteins as evidenced by the large aggregates formed when complexes were incubated for 24 hours in FBS (Fig. 3 (c) and (d)). Although cationic nanoparticles are likely to aggregate due to non-specific interaction with negatively charged proteins, incorporation of a PEG component can prevent these interactions and inhibit aggregation [21], [22]. However, in this instance the lack of effectiveness of the PEGylated CD may be due to the relatively low molecular weight of the PEG (500 Da). Indeed, in another study where we modified the surface of CD.siRNA complexes with a lipid-PEG_2000_ conjugate, aggregation in serum-containing medium was also observed, indicating that even higher PEG molecular weights of up to 5000 Da may be needed for ‘stealth’ effects [30].

Another reason for the inclusion of a PEG component is to improve the toxicity profile of the vector [21], [22]. Cationic vectors are known to cause significant toxicity, particularly to neuronal cells [43]–[45]. Here, both cationic and co-formulated complexes caused minimal toxicity, with high levels of cell viability even after 24 hours. However, a significant reduction in cell viability was seen for the co-formulated complexes at the highest molar ratio (1∶1) (Fig. 5 (b)). This may be due to the presence of some aggregates in this sample (high PDI). However, it is worth noting that the reduction in cell viability to ∼ 70% is comparable to that seen after treatment with the widely used commercial vector Lipofectamine® 2000 [17].

The incorporation of a PEG shielding layer seemed to impair interaction with the cell membrane and subsequent uptake, given that the levels of intracellular siRNA mediated by the co-formulated complexes were significantly lower than those obtained with the cationic CD alone (Fig. 4 (a)). Furthermore, the PEGylated CD had a dramatic effect on gene silencing efficiency, with no significant knockdown achieved even with the least amount of PEGylated CD in the formulation (Fig. 4 (b)), as was previously shown in non-neuronal cells (Caco2) [16]. Varying effects of PEGylation on gene knockdown have been reported by other groups, with some reporting a lack of gene silencing with a PEGylated vector [46] and others reporting highly efficient gene silencing with a PEG component in the formulation [25], [30]. In a previous study, where CD.siRNA complexes were surface modified with lipid-PEG-octaarginine, uptake levels and gene silencing were maintained compared to the unmodified complexes, likely due to the inclusion of the cell-penetrating peptide [32]. Therefore, it is clear that the effects of PEGylation on transfection depend on the vector, the PEG linker and the presence of a targeting ligand. In the current study, addition of the PEGylated CD impaired gene silencing which may be due, at least in part, to the reduction in uptake compared to the cationic CD alone. However, the levels of uptake were still greater than those achieved for naked siRNA and so other factors, such as impaired endosomal escape or intracellular release of the nucleic acid cargo, may also play a role [47].

Overall, given the higher toxicity of the 1∶1 formulation and its high polydispersity and heterogeneous size range in water, the formulation containing 10∶1 cationic CD to PEGylated CD may be more suitable for future development as a neuronal delivery vector. In order to fully optimise stability for *in vivo* use, co-formulations consisting of higher MW PEGylated CDs with a cationic counterpart warrant investigation, with a view to optimising stability in the circulation. Furthermore, the approach outlined here lends itself to the development of a CD-based formulation made specific for neuronal and CNS delivery by the attachment of a targeting ligand, such as transferrin or the RVG peptide [48], [49], via the PEG chains (‘ABCD’ type formulation). Such modifications can be facilitated by further advances in this type of CD technology.

### Conclusions

Here, we show that co-formulation is a successful approach for modifying CD-based siRNA delivery vectors for neurons. Complexes were investigated at MR20, as required for adequate neuronal siRNA transfection. Incorporation of the PEGylated CD into the formulation reduced the surface charge and improved the stability in a salt-environment, although it was not sufficient to confer serum-stability. In addition, PEGylation mediated significant effects on neuronal uptake and gene silencing, both of which were reduced due to the steric shielding effects of the PEG chains. This represents a step towards the development of a neuron-specific siRNA delivery vector, with enhances stability properties for future *in vivo* use.
